# Unraveling the direct and indirect effects of interpersonal mindfulness on school‐age bullying perpetration and victimization: The mediating role of emotional intelligence

**DOI:** 10.1002/ped4.70031

**Published:** 2025-12-22

**Authors:** Marguerita Aoun, Elias Saade, Abdallah Chahine, Georges Maalouf, Abdo Hankache, Sahar Obeid, Feten Fekih‐Romdhane, Souheil Hallit

**Affiliations:** ^1^ School of Medicine and Medical Sciences Holy Spirit University of Kaslik Jounieh Lebanon; ^2^ School of Arts and Sciences Social and Education Sciences Department Lebanese American University Jbeil Lebanon; ^3^ Department of Psychiatry “Ibn Omrane” The Tunisian Center of Early Intervention in Psychosis Razi Hospital Manouba Tunisia; ^4^ Faculty of Medicine of Tunis Tunis El Manar University Tunis Tunisia; ^5^ Applied Science Research Center Applied Science Private University Amman Jordan

**Keywords:** Adolescents, Bullying perpetration, Bullying victimization, Emotional intelligence, Interpersonal mindfulness

## Abstract

**Importance:**

As interpersonal mindfulness is a relatively recently developed concept, there is limited research on how it relates to school‐age bullying perpetration/victimization (BP/BV).

**Objective:**

To assess the direct and indirect effects of interpersonal mindfulness on school‐age BP and victimization while focusing on the mediating role of emotional intelligence.

**Methods:**

Our study utilized a cross‐sectional design between May and September 2024, and recruited 451 adolescents in schools aged between 12 and 18 years. Applying the snowball technique, a questionnaire was sent via Google Forms to the principal of each school, who redirected the questionnaire to the students after obtaining consent from their parents.

**Results:**

Emotional intelligence mediated the associations between interpersonal mindfulness and BV and BP. Higher interpersonal mindfulness was significantly associated with higher emotional intelligence. Higher emotional intelligence was significantly associated with lower BV and BP (*r* = −0.52, *P* < 0.001; *r* = −0.50, *P* < 0.001, respectively). Interpersonal mindfulness was directly associated with BP. However, no significant direct association was found with BV.

**Interpretation:**

Our results underscore the importance of promoting emotional intelligence as a core intermediate factor linking interpersonal mindfulness to bullying. Given that emotional intelligence can be regarded as a skill that can be trained, our findings can offer a potential target for prevention and intervention.

## Graphical abstract

Emotional intelligence mediated the association between interpersonal mindfulness and bullying victimization/perpetration (BV/BP). Higher interpersonal mindfulness was significantly associated with higher emotional intelligence. Higher emotional intelligence was significantly associated with lower BV/BP. Interpersonal mindfulness was not directly associated with BV but was significantly associated with lower BP.

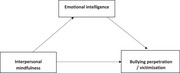

## INTRODUCTION

Adolescence represents a pivotal developmental stage, beginning with the biological transitions of puberty and culminating in the attainment of full maturity in adulthood.^1^ It is a dynamic and critical period during which various factors can influence development, steering it toward either positive or negative trajectories.^2^ Adolescence is a critical period marked by emotional challenges and maturation of the regulatory neural circuitry.^3^ During this sensitive stage, emotional challenges are strongly linked to victimization and increased risk of suicidal behavior. Higher suicide rates and significant increases have been reported outside Europe, with the highest age‐standardized suicide rate being 15.5 per 100 000 among males in the United States, with an annual increase of 3.8% among males (2009–2020) and 6.7% among females (2007–2017).^4^ It is largely spent in schools, where it facilitates negative behaviors, such as bullying, with lasting impacts on victims and perpetrators.^5^, ^6^ Bullying perpetration (BP) is a deliberate and aggressive behavior aimed at causing harm to another individual, characterized by a power imbalance in which the perpetrator holds a position of dominance over the victim, who is relatively weaker or more vulnerable.^7^ It can be either direct or indirect, with direct bullying involving physical aggression or verbal abuse, and indirect bullying involving social humiliation or exclusion.^8^ Bullying victimization (BV) is the experience of feeling persecuted by others’ acts, leading to a sense of powerlessness in stopping additional mistreatment.^9^

Children and adolescents who are victims of bullying often exhibit elevated levels of anxiety and diminished self‐confidence.^10^ Moreover, individuals who experience frequent bullying during childhood exhibit a higher frequency of adverse psychiatric consequences later in adulthood, including depression, anxiety disorders, and suicidality.^11^ Furthermore, research highlights that being a victim of school bullying and perpetrating bullying are important risk factors for negative clinical outcomes. Notably, both experiences may increase the likelihood of suicidal behavior in the short and long term.^12^ Recent systematic reviews and meta‐analyses of longitudinal studies have indicated that engaging in bullying behavior during school years is a significant predictor of increased aggression and criminal offending in later life.^13^, ^14^ A meta‐analysis conducted in 2014 estimated the prevalence of traditional bullying to be 35% and cyber‐bullying to be 15%.^15^ A recent review conducted in 2014 revealed that approximately 20%–25% of teenagers are directly involved in bullying, either as perpetrators, victims, or in dual roles as both.^16^ According to recent studies, nearly one in every four Lebanese teenagers engages in bullying, with 12% classified as offenders. Additionally, an estimated 90% of bullying incidents in Lebanon occur within school settings.^17^ Given the complex emotional dynamics involved in bullying behaviors, exploring their related factors is essential for gaining a deeper understanding of at‐risk adolescents’ profiles to enable effective early detection, timely prevention, and intervention in these phenomena. This study focused on interpersonal mindfulness as a potential factor associated with engagement in both BP and BV.

### The relationship between interpersonal mindfulness and school‐age BP/BV

Mindfulness renders individuals with the capability for attention and awareness targeted at the present moment. It fluctuates in magnitude and can be empirically evaluated separately from religious, spiritual, and cultural beliefs.^18^ Interpersonal mindfulness involves the utilization of mindfulness strategies within social contexts, prioritizing nonjudgmental acceptance, self‐awareness, and nonreactivity. Within these relationships, it encompasses attentiveness and presence.^19^ In other words, mindfulness reflects a state of intrapersonal awareness, whereas interpersonal mindfulness is built on interpersonal awareness.^20^ Although there is limited understanding of how mindfulness is expressed within the dynamic exchanges of daily interpersonal interactions,^21^ interpersonal mindfulness is suggested to render the individual the capability to not only focus on one's self, but also to pay attention to others’ needs. Interpersonal mindfulness improves interpersonal interactions and subsequently ameliorates connections with others.^20^, ^21^ Therefore, a lack of social awareness was shown to be associated with a higher rate of aggressiveness in interpersonal interactions, such as bullying others.^22^ As such, as bullying is an interpersonal problem, interpersonal mindfulness is associated with lower levels of bullying by increasing social awareness.^23^ However, as interpersonal mindfulness is a relatively recently developed concept, there is limited research on how it relates to school‐age BP and BV. This study sought to clarify this relationship by examining the role of a potential mediator, emotional intelligence.

### The mediating role of emotional intelligence between interpersonal mindfulness and school‐age BP/BV

Salovey and Mayer^24^ conceptualized emotional intelligence as a subgroup of relational intelligence, encompassing the ability to internalize and control one's own emotions as well as the emotions of others, and to use this knowledge to drive mental processes and conduct. The possible indirect role of emotional intelligence between interpersonal mindfulness and school‐age bullying was hypothesized in this study because previous evidence suggests that interpersonally mindful people are shown to be more emotionally intelligent,^25^ and that those with higher levels of emotional intelligence show a reduced risk of being bullies or victims.^26^, ^27^ Indeed, interpersonal mindfulness procures the gift of social awareness, meaning the awareness of others’ feelings and emotions. Building on the concept that emotional intelligence is the ability to comprehend other emotions,^28^ being interpersonally mindful gives us the ability to be more socially aware and therefore more emotionally aware, favoring more emotional intelligence in accordance with Basu et al.,^29^ who stated that interpersonal competence is a key component of emotional intelligence. On the other hand, individuals with high emotional intelligence levels exhibited the lowest scores on the global bullying indices and BV.^30^ In fact, bullying derives from the fact that individuals are painted with different experiences and emotions. Emotional intelligence enables interpersonal emotional comprehension and, by unifying those emotions and experiences, makes bullying behaviors less likely.^31^ Given that emotional intelligence can be regarded as a skill that can be trained,^32^ providing empirical support for the mediating role between interpersonal mindfulness and bullying can offer a potential target for prevention and intervention.

### Rationale and objectives of the present study

As documented in numerous studies on bullying in schools, bullying has become a significant concern in educational settings worldwide, with detrimental effects on adolescents’ mental health and well‐being of adolescents.^33^ *^,^* ^34^ Lebanon, like many other countries, is not immune to this issue, with its prevalence reaching up to 50% in Lebanese scholars and adolescents, who are exposed to bullying at some point.^8^ Emotional intelligence and interpersonal mindfulness are growing areas of research, with proven connections to positive social behavior.^35^ However, amidst the growing awareness of bullying, there is a notable gap in understanding the role of emotional intelligence and interpersonal mindfulness in mitigating or exacerbating bullying behaviors among Lebanese adolescents, and since emotional intelligence and interpersonal mindfulness are trainable traits,^36^ they can be enhanced through specialized training. As stated in a previous study, understanding emotional intelligence in adolescents provides us with many behavioral problem‐solving opportunities, such as bullying.^37^ In addition, another study claimed that emotional intelligence strongly favors coping mechanisms in adolescents,^38^ thus a lack of emotional intelligence will lead to negative behaviors.^39^, ^40^ By investigating these constructs within the Lebanese cultural context, this study aims to fill this gap and provide insights into creating programs aimed at fostering interpersonal mindfulness and emotional intelligence skills among adolescents. Therefore, the objective of our study was to assess the direct and indirect effects of interpersonal mindfulness on school‐age BP and BV while focusing on the mediating role of emotional intelligence. Based on prior literature, we hypothesized that (1) higher interpersonal mindfulness would be directly associated with lower BP and BV, (2) interpersonal mindfulness would be positively associated with emotional intelligence, (3) emotional intelligence would be inversely related to BP and BV, and (4) emotional intelligence would mediate the association between interpersonal mindfulness and bullying outcomes.

## METHODS

### Ethical approval

The protocol of the study was approved by the ethics committees of the Notre Dame des Secours University Hospital. Consent was written within the questionnaire and submitted online to the adolescents in schools and their parents to agree to participate in the study. All methods were performed in accordance with the relevant guidelines and regulations (in accordance with the Declaration of Helsinki).

### Study design

Our study was conducted using a cross‐sectional model between May and September 2024, using a random sample of schools from all Lebanese governorates. Twelve schools, selected using the random draw method, were approached for participation in the study, of which three declined. The schools that consented to be included were allocated as follows: two in Beirut, one in the south, four in Mount Lebanon, one in the north, and one in the Beqaa region. We contacted the principal of each school via phone calls and explained the aim and methods of our study. For those who agreed to participate, an online questionnaire, created using Google Forms software, was sent to the principal of each school and then to the students after obtaining consent from the parents and the students. Participants were chosen randomly from each school and were aged between 12 and 18 years. Students had the freedom to take part and obtained no monetary rewards. This study excluded adolescents with mental health disorders, speech problems, mental deficits, physical disabilities, chronic illnesses, illiteracy, and those who declined to participate.

### Minimal sample size

Using the equation given by Fritz and MacKinnon,^41^ we applied a small‐to‐moderate effect size of *f* = 0.26, an alpha error of 5%, a power of 80%, and 11 variables to be inserted in the model, we obtained a minimum sample size of 128.

### Questionnaire

The questionnaire was in Arabic, the native language of Lebanon, and needed 10–15 minutes to complete. The participants filled out the questionnaire via a link sent to their phones.

The questionnaire was divided into two sections. The first section consisted of sociodemographic and other characteristics, including age, gender, socioeconomic status, living situation, financial burden, sports, and social activities. Body Mass Index (BMI) was calculated based on information provided by the participants. The household crowding index was calculated by dividing the number of people living in the house by the number of rooms in the house, eliminating the bathroom and kitchen.^42^

The second part consisted of the different scales utilized:

***The Illinois Bullying Scale (IBS)***

This scale, which measures both BV (e.g., “other students called me names”) and BP (e.g., “In a group I teased other students”), was validated in Lebanon.^8^ The scale consists of 16 items subdivided into two subscales: BV, seven items, and BP, nine items. The scoring was as follows: never = 0, up to seven times or more = 4. Both subscales were used in this study. Higher scores indicate higher bullying behaviors.^43^ The current Cronbach's α value was 0.92 for BV and 0.97 for BP.

***Assessing Emotions Scale (AES)***

Validated in Lebanon,^44^ it measured emotional intelligence among participants. The scale consists of 30 items (e.g., It's easy for others to trust me), which were adapted from the following scoring system: I strongly disagree = 1, up to I strongly agree = 5. The score ranged from 30 to 150, with higher scores indicating higher emotional intelligence.^45^ The Cronbach's α value was 0.98.

***Interpersonal Mindfulness Scale (IMS‐SF‐13)***

Initially, this scale comprised 27 items, but was later condensed to 13 items. Evidence indicates that the short form has strong psychometric properties, including reliability and construct validity.^46^ Validated in Lebanon,^19^ it consists of 4 subscales: (1) Presence, (2) Awareness of Self and Others, (3) Nonjudgmental Acceptance, and (4) Nonreactivity.^47^, ^48^ The IMS‐SF‐13 yields scores ranging from 13 to 65, with higher scores reflecting a greater degree of interpersonal mindfulness.^46^ The current Cronbach's α value was 0.92.

### Statistical analysis

SPSS v.27 software was used for statistical analysis. The BP and BV scores were not considered normally distributed since the skewness and kurtosis values were between the ‐1 and +1 interval. A log transformation was applied to both scores, which showed normal distribution and were therefore used in the analysis. Student's *t*‐test was used to compare two means, analysis of variance (ANOVA) to compare three or more means, and Pearson's test to correlate two continuous variables. Mediation analysis was performed using PROCESS MACRO (SPSS add‐on) v3.4 Model 4, with the number of bootstrap samples set at 5000 and a 95% confidence interval (CI). Four routes result from this analysis: pathway a of the independent variable to the mediator, pathway b of the mediator to the dependent variable, and pathways c and c indicating the total and direct effects of the independent variable on the dependent variable. We considered mediation analysis to be significant if the confidence interval did not pass zero. Covariates entered in the model were those that showed a *P* < 0.25 in the bivariate analysis. Statistical significance was set at *P* < 0.05.

## RESULTS

In total, 451 participants participated in this study, with a mean age of 15.40 years and 53.9% being female. The other descriptive statistics for the sample are shown in Table 1.

**TABLE 1:** Sociodemographic and other characteristics of the participants (*n* = 451)

Variable	Characteristics
Sex	
Male	208 (46.1)
Female	243 (53.9)
Living situation	
Father and mother	377 (83.6)
Mother	56 (12.4)
Father	15 (3.3)
Other	3 (0.7)
School type	
Private school	420 (93.1)
Public school	31 (6.9)
Sport	
No	69 (15.3)
Fitness	74 (16.4)
Team sports	136 (30.2)
Individual sports	74 (16.4)
Outdoor activities	71 (15.7)
Artistic martial arts	27 (6.0)
Social activities	
No	255 (56.5)
Yes	196 (43.5)
Close friends	
No	184 (40.8)
Yes	267 (59.2)
Age (years)	15.40 ± 1.72
Financial burden	4.85 ± 2.29
Emotional intelligence	98.86 ± 26.69
Interpersonal mindfulness	41.69 ± 11.84
Household overcrowding index (person/room)	1.04 ± 0.45

Data are shown as *n* (%) or mean ± SD.

### Bivariate analysis of factors associated with BV and perpetration

Participants who attended public schools versus private schools (0.93 ± 0.40 vs. 0.59 ± 0.41, *P* < 0.001; 0.84 ± 0.61 vs. 0.44 ± 0.52; *P* < 0.001) and did not practice sports had significantly higher BV and BP scores (both *P* < 0.001). Moreover, male gender, living with the father, and having close friends were significantly associated with higher BP scores. However, the participants who did not have close friends had significantly higher BV scores (Table 2). Furthermore, higher household overcrowding index and financial burden were significantly associated with higher BV score, whereas higher emotional intelligence (*r* = −0.52, *P* < 0.001; *r* = −0.50, *P* < 0.001) and interpersonal mindfulness (*r* = −0.34, *P* < 0.001; *r* = −0.58, *P* < 0.001) were significantly associated with lower BV and BP scores (Table 3).

**TABLE 2:** Bivariate analyses of factors associated with bullying victimization and perpetration scores

	Bullying victimization			Bullying perpetration		
Variables	Score	*P‐*value	Effect size	Score	*P‐*value	Effect size
Sex		0.473	0.088		<0.001	0.342
Male	0.64 ± 0.43			0.57 ± 0.57		
Female	0.61 ± 0.42			0.39 ± 0.50		
Living situation		0.317	0.013		0.027	0.020
mother	0.61 ± 0.43			0.45 ± 0.52		
Mother	0.65 ± 0.38			0.54 ± 0.57		
Father	0.80 ± 0.37			0.82 ± 0.70		
Other	0.97 ± 0.32			0.10 ± 0.17		
School type		<0.001	0.823		<0.001	0.738
Private school	0.59 ± 0.41			0.44 ± 0.52		
Public school	0.93 ± 0.40			0.84 ± 0.61		
Sport		<0.001	0.137		<0.001	0.065
No	0.96 ± 0.34			0.67 ± 0.64		
Fitness	0.57 ± 0.34			0.50 ± 0.53		
Team sports	0.60 ± 0.43			0.49 ± 0.54		
Individual sports	0.55 ± 0.43			0.41 ± 0.52		
Outdoor activities	0.56 ± 0.40			0.21 ± 0.33		
Artistic martial arts	0.43 ± 0.34			0.64 ± 0.53		
Social activities		0.262	0.139		0.163	0.131
No	0.65 ± 0.42			0.50 ± 0.56		
Yes	0.59 ± 0.43			0.43 ± 0.51		
Close friends		<0.001	0.139		<0.001	0.131
No	0.74 ± 0.44			0.31 ± 0.44		
Yes	0.52 ± 0.37			0.58 ± 0.58		

The Log of bullying victimization and perpetration scores were used.

**TABLE 3:** Pearson correlation matrix

Variables	Bullying victimization	Bullying perpetration	Age	Household overcrowding index	Financial burden	Emotional intelligence	Interpersonal mindfulness
Bullying victimization	1						
Bullying perpetration	0.07	1					
Age	0.01	−0.01	1				
Household overcrowding index	0.12^*^	0.06	−0.04	1			
Financial burden	0.23^***^	0.01	−0.02	0.30^***^	1		
Emotional intelligence	−0.52^***^	−0.50^***^	−0.02	−0.06	−0.09	1	
Interpersonal mindfulness	−0.34^***^	−0.58^***^	−0.01	−0.03	−0.03	0.72^***^	1

* *P* < 0.05; ^**^ *P* < 0.01; ^***^ *P* < 0.001. The Log of bullying victimization and perpetration scores were used.

### Analysis of mediation

**FIGURE 1:**
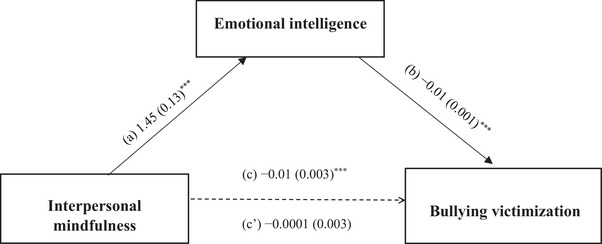
(a) Relation between interpersonal mindfulness and emotional intelligence (*R* ^2^ = 0.436); (b) Relation between emotional intelligence and bullying victimization (*R* ^2^ = 0.374); (c) Total effect of interpersonal mindfulness on bullying victimization (*R* ^2^ = 0.269); (c’) Direct effect of interpersonal mindfulness on bullying victimization. The numbers represent regression coefficients and their standard errors. ^***^ *P* < 0.001. The Log of the bullying victimization score was used.

The mediation analysis, taking the Log BV score as the dependent variable, was adjusted for the following covariates: school type, close friends, sports, household overcrowding index, and financial burden. Emotional intelligence (indirect effect: Beta = −0.011; Boot SE = 0.002; Boot 95% CI: −0.015, −0.007) fully mediated the association between mindfulness and BV. Higher interpersonal mindfulness was significantly associated with higher emotional scores. Higher emotional intelligence was significantly associated with a lower BV score. Interpersonal mindfulness was not directly associated with BV (Figure 1).

**FIGURE 2:**
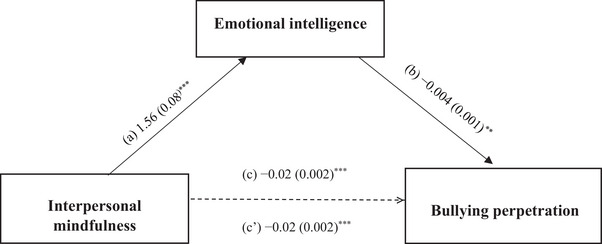
(a) Relation between interpersonal mindfulness and emotional intelligence (*R* ^2^ = 0.559); (b) Relation between emotional intelligence and bullying perpetration (*R* ^2^ = 0.445); (c) Total effect of interpersonal mindfulness on bullying perpetration (*R* ^2^ = 0.432); (c’) Direct effect of interpersonal mindfulness on bullying perpetration. The numbers represent regression coefficients and their standard errors. ^**^ *P* < 0.01; ^***^ *P* < 0.001. The Log of the bullying perpetration score was used.

Mediation analysis, taking the Log BP score as the dependent variable, was adjusted for the following covariates: school type, close friends, sport, living situation, sex, and social activities. Emotional intelligence (indirect effect: Beta = −0.055; Boot SE = 0.002; Boot 95% CI: −0.009, −0.002) partially mediated the association between interpersonal mindfulness and BP. Higher interpersonal mindfulness was significantly associated with higher emotional intelligence and directly associated with lower BP score. Higher emotional intelligence was significantly associated with lower BP score (Figure 2).

## DISCUSSION

The present study showed that emotional intelligence fully mediated the association between interpersonal mindfulness and BV, and partially mediated the association between interpersonal mindfulness and BP. Specifically, higher interpersonal mindfulness was largely linked to greater emotional intelligence, which in turn was associated with lower BV and BP. Interpersonal mindfulness was directly related to BP but not to BV. To our knowledge, this study is the first to explore this mediating pathway among adolescents in schools.

In our study, a higher interpersonal mindfulness was directly associated with BP but not with BV. In accordance with our findings, it has been suggested that school‐based mindfulness interventions can help reduce bullying behaviors through the improvement of self‐control and trait mindfulness.^49^ A study conducted in Kashan town with elementary students revealed that enhancing mindfulness reduces bullying behaviors, such as excluding children from friend groups and hurtful humor.^50^ Therefore, mindfulness strategies can serve as a proactive approach to enhance student well‐being.^51^ It can be hypothesized that this association is influenced by the fact that mindfulness nurtures a peaceful behavior based on empathy,^52^ understanding others’ feelings and perceptions, and treating them as equals while solving an issue in a collaborative manner. This behavior encourages mutual respect and clear communication, thus reducing the urge to resolve aggressive responses and impose dominance. Finally, the absence of a direct association between interpersonal mindfulness and BV might arise from the inability to completely eliminate external factors, such as group hierarchies, subjecting even a mindful person to BV.

Our hypothesis was supported, with emotional intelligence acting as a significant mediator between interpersonal mindfulness and BV and as a partial mediator between interpersonal mindfulness and BP. A higher interpersonal mindfulness was associated with greater emotional intelligence, which in turn was associated with lower BV and BP. Our finding that interpersonal mindfulness is positively linked to emotional intelligence is in agreement with the literature. While a meta‐analysis has already proven a relationship between mindfulness and emotional intelligence,^53^ our research is the first to demonstrate that higher interpersonal mindfulness is associated with higher emotional intelligence. Previous studies have shown that mindfulness training in healthcare workers provides psychological care by reducing stress, anxiety, and depression.^54^, ^55^ Likewise, mindfulness training has been proven to improve emotional intelligence skills through emotional regulation, expression, assimilation, and self‐compassion.^25^, ^56^ Brown and Ryan^57^ stated that mindfulness training improves the capacity to clearly depict and understand one's own emotional states. A plausible explanation for this association is that true interpersonal mindfulness translates not only into recognizing one's emotions but also understanding the reason behind them. Whether the cause is a present complication, a past misunderstanding, a heavy train of thoughts, or repercussions of a certain trauma, the person is able to link it all together, pause, and consciously control his reactions.^58^ Once a person is deeply familiarized with his/her emotions, it becomes easier to perceive and understand others’ emotions, to actually be able to “read the room,” and to put him/herself in “someone else's shoes.” This would effectively strengthen their social dynamics and contribute to positive relationships.

In our study, a higher emotional intelligence was widely associated with lower BV and BP. These results are in agreement with prior studies showing that victims of bullying were less capable of understanding one's emotions appropriately and handling others’ emotions.^26^, ^59^ According to Lomas et al.,^60^ adolescents with limited ability to understand others’ emotions may struggle to identify the consequences of their actions, making it difficult for them to grasp the harmful effects of their bullying behavior on others. Adolescents with more emotional stability have better control over negative behaviors such as anger or aggressiveness due to the mediating effect of emotional intelligence.^61^, ^62^ This relation might derive from the fact that a distinctive characteristic of a highly emotionally intelligent individual is the ability to empathize with others and comprehend their emotions and reactions,^63^ thus allowing the individual to resolve any conflict in a constructive manner using good communication and negotiation, and not engaging in bullying patterns. Moreover, owing to the strong power of emotional control, the individual is able to set boundaries and navigate any negative or aggressive behaviors,^64^ minimizing their negative repercussions on their well‐being and mental health.

### Limitations

Our study has several limitations that have to be pinpointed. First, recall bias: this study depends on the adolescents filling a questionnaire; some respondents may not recall certain events properly, so they may under‐ or over‐report some information. Second, our findings cannot be interpreted as evidence of causal association due to the cross‐sectional nature of our study design. Therefore, future studies should focus on reproducing this study using longitudinal data. Third, our study may have been subject to selection bias because of the application of the snowball technique for data collection. Furthermore, our sample consisted mainly of private schools, which means that we must acknowledge the potential effect of private schools on the variables, limiting the generalizability of our findings. Fourth, our study relied exclusively on self‐reported measures, which may have introduced social desirability or response bias. Fifth, although we focused on emotional intelligence and interpersonal mindfulness, other relevant psychosocial variables (e.g., family support, peer relationships, and socioeconomic background) were not assessed and may have confounded the observed associations. Finally, cultural factors specific to Lebanon may limit the external validity of our findings, and replication in different cultural and educational contexts is warranted.

### Clinical implications

Our study offers meaningful insights into the mediation of emotional intelligence among interpersonal mindfulness, BV, and BP. Bullying is on the rise, especially in adolescents in schools, reaching the Middle East, 20% in the United Arab Emirates, and 44.2% in Jordan, while in Lebanon it attained 33.6%.^65^ Bully victims in schools are at a higher risk of mental health disorders and substance addiction.^66^, ^67^ Therefore, implementing targeted strategies to safeguard against bullying is crucial for protecting adolescents. In our study, we found that high interpersonal mindfulness scores were significantly associated with greater emotional intelligence, which in turn was associated with reduced BV and BP scores. Schools should focus on establishing programs to reinforce emotional intelligence and interpersonal mindfulness training, as they significantly reduce BV and BP scores. In fact, implementing an emotional intelligence‐based program in schools improved students’ behavioral aspects.^68^ Another six‐step program developed in 2004 proved to be efficient in improving emotional intelligence skills in middle school.^69^ Furthermore, a previous meta‐analysis focused on implementing programs that target bystanders/peers (e.g., students and teachers) who are witnessing bullying behaviors and are not intervening.^70^ In fact, it has been proven that eliminating bystanders’ neutral behavior, considered as support for bullying, and encouraging intervening measures helps reduce bullying actions.^71^

### Conclusion

Taken together, the main point of this study is that interpersonal mindfulness exerts its protective effects on bullying outcomes, largely through the enhancement of emotional intelligence. Our results showed that emotional intelligence mediates the association between interpersonal mindfulness and BP/BV. High interpersonal mindfulness was linked to higher emotional intelligence, which in turn was linked to reduced levels of BV and BP in adolescents. Our findings have three key implications. First, they underscored the importance of promoting interpersonal mindfulness and emotional intelligence as core intermediate factors linking interpersonal mindfulness to bullying. Second, school‐based programs should incorporate both interpersonal mindfulness and emotional intelligence training as part of standard curricula, not only to reduce bullying behaviors but also to foster broader socio‐emotional skills that enhance adolescent well‐being. Third, they highlighted that such interventions may have long‐term benefits, potentially reducing the risk of psychiatric problems and improving resilience into adulthood. Although our sample was drawn from Lebanese schools, the mechanisms linking interpersonal mindfulness, emotional intelligence, and bullying are likely cross‐culturally relevant. Additional experimental research should explore how the integration of emotional intelligence and interpersonal mindfulness in adolescent education can reduce bullying and potentially ameliorate behavior and mental health in adulthood.

## CONFLICT OF INTEREST

The authors declare no conflict of interest.
